# Social Support From Kin and Mortality Risk Among Older Adults in a Historical Aging Population

**DOI:** 10.1093/geroni/igaa057.1337

**Published:** 2020-12-16

**Authors:** Julia Jennings

**Affiliations:** University at Albany, SUNY, Albany, New York, United States

## Abstract

Family are often sources of social, instrumental, and financial support for older adults. However, in many types of survey and archival data, details on the provision of support are lacking. This study examines the association between kin availability and cause-specific mortality among adults over age 60 using multiple longitudinal linked data sources from North Orkney, Scotland, 1851-1911. This study explores the relationships between cause of death and kin availability, as certain ailments may be amenable to interventions related to social support in this period while others may not. This approach will aid in interpreting the effects of social support that may be transmitted through kin networks. Reconstructed individual life courses (N=4,946) and genealogies, in combination with data on the proximity non-coresident kin, are used to examine kin availability and propinquity over the life course. Cause of death is available from death records and has been coded into the ICD. Orkney provides an interesting case study as longitudinal information is available on mortality and kin availability during a time of population aging. Kin availability is associated with longevity in this sample, while cause-specific analysis allows us to evaluate the role of social support in promoting longevity net of this association.

